# Changes in the Epidemiology and Causative Pathogens of Meningitis in Children After the Outbreak of the Coronavirus Disease 2019: A Multicenter Database Study

**DOI:** 10.3389/fped.2022.810616

**Published:** 2022-04-14

**Authors:** Jooyoung Lee, Arum Choi, Kyunghoon Kim, Joong Hyun Bin, Tae Hoon Eom, Il Han Yoo, Da Hye Yoon, Sukil Kim, Young Hoon Kim

**Affiliations:** ^1^Department of Pediatrics, College of Medicine, The Catholic University of Korea, Seoul, South Korea; ^2^Department of Preventive Medicine and Public Health, College of Medicine, The Catholic University of Korea, Seoul, South Korea; ^3^Department of Pediatrics, Seoul National University College of Medicine, Seoul, South Korea

**Keywords:** COVID-19, meningitis, children, childhood meningitis, neuroinfectious disease

## Abstract

**Background:**

With the outbreak of the COVID-19 pandemic, non-pharmaceutical interventions such as social distancing have been implemented worldwide, and a decrease in other infectious diseases has been reported as an unexpected benefit. However, to date, studies are lacking regarding the effects of the COVID-19 pandemic on neuroinfectious diseases; therefore, we aimed to determine the effects of the COVID-19 pandemic on the incidence of meningitis, which is the most common infectious disease in children.

**Methods:**

This retrospective study used electronic medical record data from five university hospitals located in the metropolitan cities in Korea. This study included patients aged <18 years who were diagnosed with meningitis between January 2017 and December 2020. We analyzed the clinical characteristics of patients with meningitis and changes in the incidence and causative pathogens of meningitis before and after the COVID-19 outbreak.

**Results:**

The study included 677 patients with meningitis. Following the outbreak of COVID-19 in Korea in January 2020, the incidence of childhood meningitis significantly decreased and seasonal changes noted yearly disappeared. There was a difference in the age distribution of patients with meningitis. The incidence of meningitis decreased significantly in children aged >5 years, and the incidence in children <5 years of age relatively increased (*p* < 0.001). In addition, there was a notable decrease in the cases of suspected meningitis (*p* < 0.001). The incidence of enteroviral meningitis, the most common cause of meningitis, significantly decreased.

**Conclusion:**

After the COVID-19 outbreak, the incidence of childhood meningitis significantly decreased with the implementation of non-pharmaceutical interventions. Absence of enteroviral meningitis and decrease in the proportion of patients aged ≥5 years with meningitis having mild symptoms were noted. Consequently, it can be concluded that the non-pharmaceutical interventions (NPIs) instituted to prevent the spread of COVID-19 had some effect on reducing the incidence of meningitis.

## Introduction

Meningitis is the most common infectious disease of the central nervous system (CNS). Approximately 2.8 million people worldwide suffer from meningitis yearly; meningitis is usually prevalent in children, especially under the age of 5 years (1, 2). In Korea, approximately 9,000 children are affected by meningitis annually (3).

Since the outbreak of the COVID-19 pandemic in 2019, many countries have sought to prevent the spread of COVID-19 by initiating non-pharmaceutical interventions (NPIs), such as mask-wearing, hand hygiene, and social distancing, which can reduce the spread of infectious diseases (4). Direct attendance at the kindergartens and schools was suspended for a long time.; thereby reducing the possibility of infection transmission by direct contact, droplets, or airborne, and this situation paradoxically demonstrates an unexpected benefit of the COVID-19 NPIs (5).

The incidence of infectious diseases in children has decreased significantly since the COVID-19 outbreak. The decreases in respiratory diseases, such as acute otitis media, bronchiolitis, common cold, group, influenza, and pneumonia, have been notable, and cases of gastroenteritis and urinary tract infection have also decreased (6). Hospital visits and hospitalization in children have also decreased owing to the reduction in pediatric infectious diseases (7). In Korea, the spread of the common respiratory virus was suppressed by social distancing, with the impact being greater when more stringent social distancing was implemented, (8).

Studies evaluating the impact of NPI implementation on the incidence of infectious CNS diseases after the COVID-19 outbreak are insufficient. Thus, we aimed to determine the effects of the outbreak of COVID-19 on changes in the characteristics of meningitis, which is the most common CNS infection among children. In this study, we investigated the changes in the incidence, causative pathogens, and age of onset of childhood meningitis before and after the COVID-19 outbreak.

## Materials and Methods

### Data Sources and Study Population

This was a retrospective study using data extracted from the clinical data warehouse of five hospitals (Seoul St. Mary’s Hospital, Yeouido St. Mary’s Hospital, Uijeongbu St. Mary’s Hospital, Bucheon St. Mary’s Hospital, and St. Vincent’s Hospital), which are in areas with a population of over 800,000 people each.

We extracted data for patients aged <18 years who were diagnosed with meningitis based on the International Statistical Classification of Diseases and Related Health Problems 10th Revision code between January 2017 and December 2020. The diagnostic codes used for extraction are summarized in the Supplement. We identified the patient’s sex, age, CSF examination, hospitalization, medication, and underlying disease through a review of the electronic health records.

**FIGURE 1:**
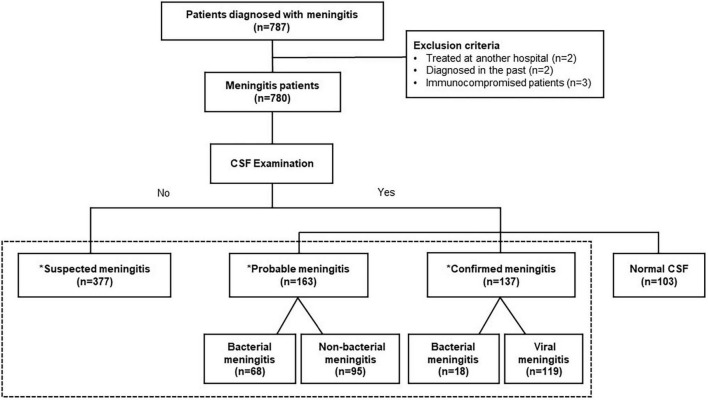
Flowchart of the study population. *Classified by reference to the WHO’s IB-VPD (invasive bacterial vaccine preventable diseases) surveillance network’s meningitis definition criteria. CSF, cerebrospinal fluid.

Patients who were diagnosed with meningitis but treated in other hospitals, lost to follow-up, not newly diagnosed between January 2017 and December 2020, or with immunodeficiency were excluded from the study. Patients with normal cerebrospinal fluid (CSF) findings were also excluded (Figure 1).

We categorized patients diagnosed with meningitis as suspected/probable/confirmed meningitis, depending on the CSF and causative pathogen findings (9). We also checked for changes in diagnosis before and after the COVID-19 outbreak.

### Outcome Measure

The main outcome of this study was the change in the incidence of meningitis before and after the COVID-19 outbreak. The secondary outcome was the change in the age distribution of meningitis. In addition, we compared the causative organisms of meningitis before and after COVID-19 to identify the relationship between changes in the causative pathogen of meningitis and changes in the incidence rate of meningitis. We aimed to identify the proportion of enteroviral meningitis in all meningitis cases. Using the sentinel surveillance infection data of the Korea Disease Control and Prevention Agency, we identified the number of patients in Korea with enteroviral meningitis between January 2017 and December 2020.

### Statistical Analysis

We evaluated the differences in sex, age, CSF study results, blood culture, treatment, admission, and diagnosis before and after the COVID-19 outbreak. The Pearson’s chi-squared test and Fisher’s exact test were used to analyze the categorical variables, while the Mann-Witney U test was used to analyze non-normal distribution data. We used the automatic selection of the ETS (error, trend, seasonal) model (10) to calculate the predicted incidence in 2020, and set it to capture different components (error, trend, and season) of a time series. We evaluated the difference between predicted and actual incidences using the chi-squared test. R version 4.0.0 (R Foundation for Statistical Computing, Vienna, Austria) was used for all statistical analyses.

### Ethics Statement

Our study was approved by the Institutional Review Board of the Catholic University of Korea (XC21WIDI0061). This study was exempted from the requirement for informed consent owing to its retrospective nature.

## Results

### Demographics and Characteristics of the Study Population

The study population was divided into two groups based on the outbreak of COVID-19 before and after the outbreak and their demographics and characteristics are summarized in Table 1. A total of 677 patients with meningitis were included in this study. There were 644 patients between 2017 and 2019, and 30 patients in 2020 after the outbreak of COVID-19, showing a significant decrease in the number of patients per year. There was a significant difference in the age distribution of patients with meningitis before and after the outbreak with more than half of patients aged ≥5 years between 2017 and 2019, while the proportion of patients aged 1 month to 1 year significantly increased after the outbreak. There was also a difference in the performance of CSF tests between the two groups (*p* < 0.001). There was also a significant difference in the distribution of meningitis classifications before and after the outbreak (*p* < 0.001). Suspected meningitis accounted for more than half of all meningitis cases between 2017 and 2019, but the proportion of probable meningitis cases increased by more than 50% in 2020. In addition, the proportion of confirmed meningitis was also notably different before and after the outbreak (21.02% vs. 3.33%). For confirmed meningitis, the causative pathogen of meningitis was mainly viruses before the outbreak, but no patients were identified with viral meningitis, and only one patient was diagnosed with bacterial meningitis after the outbreak. The ratio of meningitis patients treated with antibiotics was also different between the two groups. Between 2017 and 2019, three-quarters of patients diagnosed with meningitis were treated with antibiotics, but in 2020, antibiotics were used in all patients (*p* < 0.05).

**TABLE 1:** The demographic and clinical characteristics of the study population before and after the COVID-19 outbreak.

Total no. of patients (*n* = 677)	2017–2019 (*n* = 647)	2020 (*n* = 30)	*p*-value
**Sex**			
Male	397 (61.36)	16 (53.33)	0.49*
**Age**			
0–30 days	24 (3.71)	1 (3.33)	**<0.001** *
31 days–12 months	113 (17.47)	16 (53.33)	
13 months–5 years	102 (15.77)	2 (6.67)	
>5 years	408 (64.06)	11 (36.67)	
**CSF study**	277 (42.81)	23 (76.67)	**<0.001** *
**Classification**			**<0.001** *
Suspected meningitis	370 (57.19)	7 (23.33)	
Probable meningitis	141 (21.79)	22 (73.33)	
Confirmed meningitis	136 (21.02)	1 (3.33)	
Viral meningitis	119 (87.50)	0 (0)	
Bacterial meningitis	17 (12.50)	1 (100)	
**Hospitalization**	594 (91.81)	29 (96.67)	0.5**
**Treatment**			
Antibiotics	499 (77.13)	30 (100)	**<0.05** *
Antiviral agents	29 (4.48)	0 (0)	NA
Systemic corticosteroid	13 (2.01)	2 (6.67)	NA

*All variables are presented as number (%).*

*Chi-squared test, **Fisher’s Exact Test.

*COVID-19, coronavirus disease 2019, CSF, cerebrospinal fluid, NA, not available.*

*Bold values indicate p < 0.05.*

### Trends in the Monthly Incidence of Meningitis

**FIGURE 2:**
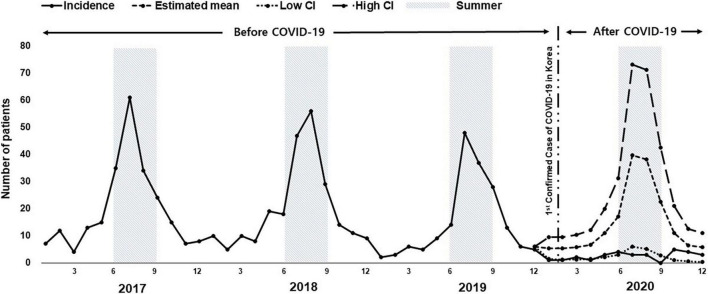
Changes in the incidence of meningitis from January 2017 to December 2020. The solid line shows observed data. The dashed line shows the estimated value without the coronavirus disease 2019 (COVID-19) pandemic. The long-dashed line and dotted line show high and low confidence intervals (CIs), respectively (ETS [error, trend, seasonal] model). The shaded areas between June and September correspond to summer. The start of the COVID-19 outbreak in Korea is indicated by the dash-double dotted line.

The monthly incidence of meningitis, including suspected, probable, and confirmed meningitis diagnosed between January 2017 and December 2020, is shown in Figure 2. Prior to the COVID-19 outbreak, there were seasonal changes in the number of meningitis patients, with a marked increase in meningitis cases between June and September in each summer. However, in 2020, after the outbreak, the seasonal change in the incidence of meningitis disappeared. Considering the existing incidence rate of meningitis, the estimated incidence pattern of meningitis in 2020 was calculated with the assumption that there was no COVID-19 pandemic and compared to the actual incidence pattern of meningitis. The results showed that the incidence of meningitis was significantly lower after the outbreak than that estimated in 2020 (*p* < 0.001).

### Characteristics of Patients Who Underwent Cerebrospinal Fluid Examination Before and After the Coronavirus Disease 2019 Outbreak

The characteristics of the patients who underwent CSF examinations (patients with probable or confirmed meningitis) before and after the COVID-19 outbreak are summarized in Table 2. There were no significant differences in the sex ratio, age distribution, and CSF test results (white blood cell count, protein, and glucose). Most patients were treated with antibiotics, regardless of the cause of meningitis or CSF results.

**TABLE 2:** The characteristics of the patients that underwent CSF examination before and after the COVID-19 outbreak.

Total patients (*n* = 300)	2017-2019 (*n* = 277)	2020 (*n* = 23)	*p*-value
**Sex**			
Male	180 (64.98)	13 (56.52)	0.56*
**Age**			
0–30 days	22 (7.94)	1 (4.35)	0.13**
31 days–12 months	110 (39.71)	15 (65.22)	
13 months–5 years	30 (10.83)	2 (8.70)	
>5 years	115 (41.52)	5 (21.74)	
**CSF study**			
WBC count (cells/μL)			
0–9	52 (18.77)	1 (4.35)	0.14**
10–99	113 (40.79)	14 (60.87)	
100–999	97 (35.02)	8 (33.33)	
≥1000	15 (5.42)	0 (0)	
Protein (mg/dL)	53.6 [35.1;84.3]	72.0 [49.0;104.5]	**<0.05^+^**
Glucose, (mg/dL)	60.0 [52.0;68.0]	58.0 [50.5;64.5]	0.583^+^
**Causative pathogen of meningitis**			
Bacteria			
Bacterial culture (CSF)	13 (4.69)	0 (0)	
Bacterial culture (Blood)	13 (2.01)	1 (3.33)	
Latex agglutination test (CSF)	4	0	
Virus			
Enterovirus RT-PCR (CSF)	116	0	
HSV type I PCR (CSF)	1	0	
Varicella zoster virus PCR (CSF)	2	0	
**Hospitalization period, days**	5.0 [4.0; 7.0]	7.0 [4.0;10.5]	**<0.05^+^**
**Treatment**			
Antibiotics	263 (94.95)	23 (100)	0.56*
Antiviral agents	27 (9.74)	0 (0)	NA
Systemic corticosteroid	12 (4.33)	1 (4.17)	NA

*Discrete variables are presented as number (%).*

*Chi-squared test, **Fisher’s Exact Test, ^+^Mann–Whitney U test.

*Continuous variables are presented as median [Q1; Q3].*

*CSF, cerebrospinal fluid, COVID-19, coronavirus disease 2019, NA, not available, PCR, polymerase chain reaction, RT, reverse transcription, WBC, white blood cell.*

*Bold values indicate p < 0.05.*

### Changes in Pathogens Causing Meningitis

**FIGURE 3:**
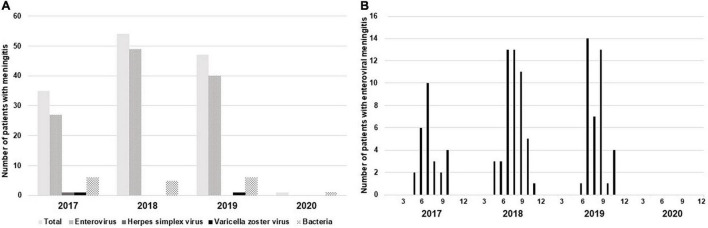
Causative organisms of meningitis. **(A)** Changes in the causative organisms of confirmed meningitis are expressed yearly. **(B)** Monthly distribution pattern of enterovirus, which is the most common cause of confirmed meningitis.

In this study, the causative pathogens of meningitis were identified in 137 patients. According to the annual rate of the causative pathogens of meningitis, before the COVID-19 outbreak, enterovirus was the most common cause of meningitis, and there was no significant difference in the proportion of enterovirus among all the causative organisms (Figure 3A). According to the monthly incidence of enteroviral meningitis, as shown in all the patients with meningitis in Figure 2, there was seasonal variation, and the incidence of enteroviral meningitis was higher, especially in the summer (Figure 3B). However, no case of enteroviral meningitis was noted after the outbreak. Prior to the outbreak, the percentage of bacterial meningitis among the confirmed meningitis patients was 12.5%. The most common pathogens of bacterial meningitis were *Streptococcus agalactiae* (*n* = 5) and *Escherichia coli* (*n* = 5); however, only one patient was confirmed to have bacterial meningitis caused by *Streptococcus agalactiae* after the outbreak.

**FIGURE 4:**
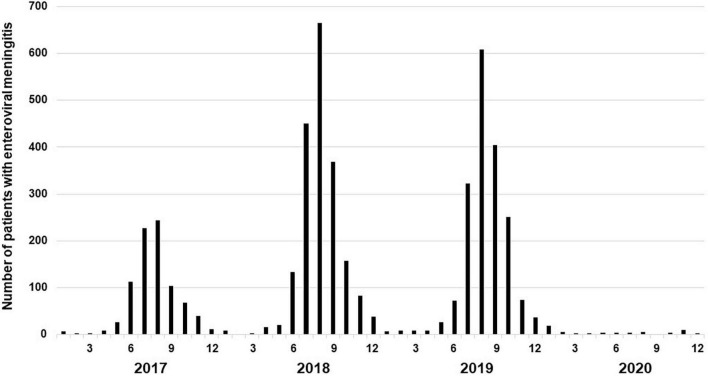
Monthly frequency distribution of enteroviral meningitis included in the sentinel surveillance system of the Korea Disease Control and Prevention Agency (KDCA) between January 2017 and December 2020.

In order to verify that the change in the prevalence of enteroviral meningitis before and after the outbreak in our study showed the same trend as the national data, we confirmed the monthly incidence of enteroviral meningitis in Korea in our study (Figure 4). From January 2017 to December 2019, the incidence of enteroviral meningitis showed seasonal variations, with the highest incidence every summer. However, after the COVID-19 outbreak, the incidence of enterovirus meningitis significantly decreased with no seasonal variations in both the national data and our study.

## Discussion

### Summary of the Results

Since the outbreak of COVID-19, the incidence of meningitis in children has decreased significantly, and the seasonal variation of meningitis before the outbreak has disappeared. The incidence of meningitis in children aged >5 years has decreased, and the incidence of meningitis in infants has relatively increased. After the outbreak, the proportion of suspected meningitis significantly decreased, and the cases of enterovirus meningitis, which was the most common cause of childhood meningitis before the outbreak, were significantly reduced.

### Differences Compared to Previous Studies

Some studies have been conducted on the changes in the incidence of meningitis after the COVID-19 outbreak (11–14). However, previous studies only analyzed changes in the incidence of meningitis caused by specific bacteria or viruses in the entire population. Most of these studies showed a decrease in the incidence of enteroviral meningitis and a change in the incidence of invasive meningococcal meningitis after the outbreak in the entire population. However, in this study, we confirmed changes in the overall incidence of meningitis in children before and after COVID-19, regardless of the causative pathogen of meningitis. This study is different because it included both patients diagnosed with meningitis *via* clinical symptoms alone (suspected meningitis) and patients who underwent CSF examination (probable meningitis) or who had causative pathogens of meningitis identified (confirmed meningitis).

### New Findings in This Study

#### Change in the Causative Pathogen of Meningitis

Enterovirus is a common cause of meningitis (15, 16). In this study, enterovirus was the most common pathogen in confirmed meningitis in children before the COVID-19 outbreak. However, no case of enteroviral meningitis was confirmed, and there were no seasonal variations after the outbreak. These results are similar to those of previous studies that reported a decrease in the incidence of enteroviral meningitis and the disappearance of seasonal changes following the outbreak (11–13). This fact seems more evident in that it shows the same results not only in the patients included in our study but also in the national prevalence data before and after the outbreak (Figure 4).

After the COVID-19 outbreak, the number of bacterial meningitis cases decreased significantly. However, we found that there was no significant difference in the proportion of patients with confirmed bacterial meningitis to all the patients with meningitis before and after the COVID-19 outbreak (before outbreak: 17/647 = 2.63%, after outbreak: 1/30 = 3.33%). Although we attempted to confirm whether there was a change in the distribution of the pathogens causing bacterial meningitis, the change could not be confirmed because the number of patients with bacterial meningitis after the COVID-19 outbreak was too small.

In conclusion, the decline in the incidence of enteroviruses had a significant impact on the overall decrease in the incidence of childhood meningitis. The effects of the changes in the incidence of bacterial meningitis on the change in the incidence of all childhood meningitis was insignificant.

#### Change in the Age of Onset

In addition to the decrease in the incidence of enteroviral meningitis, another prominent change noted after the COVID-19 outbreak in this study was the age of onset. After the outbreak, the number of patients decreased in children aged ≥5 years and <5 years, respectively. However, there was a difference in the distribution of the onset age before and after the COVID-19 outbreak. Before the outbreak, meningitis accounted for more than half of all the cases in children aged ≥5 years. However, after the outbreak of COVID-19, the rate of meningitis in children aged ≥5 years decreased (36.67%); however, the rate of meningitis in children aged <5 years increased (before outbreak: 36.95%, after outbreak: 63.33%). Children aged <5 years had a relatively less reduction in the incidence of meningitis than children aged ≥5 years. It is presumed that this change is related to the change in the ratio of suspected, probable, and confirmed cases of meningitis. After the COVID-19 outbreak, the decrease in the number of patients with suspected meningitis was noticeable. Among the patients with suspected meningitis before the COVID-19 outbreak, the proportion of children aged ≥5 years was more than twice as high as that of children aged <5 years [≥5 years: 294/408 (72.1%) vs. <5 years: 76/239 (31.8%)]. Therefore, since the number of patients with meningitis aged ≥5 years with a relatively high proportion of suspected meningitis decreased significantly, it resulted in an increase in the proportion of the incidence of meningitis in children aged <5 years after the COVID-19 outbreak.

#### Decreasing Pattern of Mild Meningitis

There was a difference in the classification of meningitis before and after the COVID-19 outbreak. Before the outbreak, suspected meningitis accounted for more than half of the cases of meningitis, but after the outbreak, the proportion of suspected meningitis decreased; instead, the proportion of probable/confirmed meningitis increased. In addition, there was a difference in the proportion of patients that used antibiotics before and after the outbreak. Before the outbreak, a quarter of the patients did not receive antibiotics, but after the outbreak, all the patients received antibiotic treatment. The patients with suspected meningitis usually had mild symptoms, and the percentage of patients who did not receive antibiotic treatment was significantly higher than that of those with probable or confirmed meningitis. Generally, because meningitis with mild symptoms is suspected to be viral meningitis (16–18), supportive care is often performed without antibiotic treatment or CSF examination. Although several studies, including this study, have confirmed that the incidence of enteroviral meningitis, which is the most common cause of meningitis, has decreased, it is difficult to find studies that have reported changes in the incidence of bacterial meningitis. Considering these points, it can be assumed that the incidence of meningitis with mild symptoms decreased after the outbreak or patients with mild symptoms had decreased visits to the hospital owing to the fear of contracting the COVID-19.

### Social Distancing in Korea and Meningitis

In Korea, national social distancing began on March 22, 2020, as the number of patients with COVID-19 increased nationwide following the first outbreak of COVID-19 on January 20, 2020, in adults and February 18, 2020, in childhood (19). Social distancing in Korea was largely divided into three stages according to the average number of daily confirmed COVID-19 cases in Korea per week. Basically, mask-wearing, hand hygiene, maintaining physical distancing, managing the list of visitors, ventilation and disinfection were performed at all the stages. In addition, measures such as limiting the number of private gatherings, limiting the number of students attending school, limiting the operation of public facilities, or limiting the number of participants in religious activities were taken at each stage (20).

Social distancing caused by the COVID-19 outbreak reduced the incidence of meningitis in children, and the seasonal variation disappeared. Among the cases with meningitis, the decrease in viral meningitis, especially enteroviral meningitis, was noticeable. Through this result, it is thought that social distancing played a role in reducing viral meningitis. Social distancing did not have a significant effect on the occurrence of bacterial meningitis, but attention should be paid when interpreting these results because the number of patients with meningitis identified after the outbreak of COVID-19 was too small.

### Limitations

This study has several limitations. First, this was a retrospective study that used electronic medical record data. Since the data used in this study were provided anonymously, it is possible that some patients were included more than once. Second, our study analyzed patients from five university hospitals located in the Korean metropolitan area. Since the COVID-19 outbreak in Korea showed different patterns depending on the region, it may be thought that the results of this study do not fully reflect the nationwide situation. However, we conducted this study using data from five hospitals located in large cities where approximately half of the Korean population lives. Therefore, we judged that this was quite representative. From our results, the decrease in the incidence of enteroviral meningitis was the same as that of the entire Korean data, indicating indirectly that this study was representativeness.

## Conclusion

This study identified a decrease in meningitis in children after the outbreak of COVID-19, especially meningitis with mild symptoms in children aged >5 years. The absence of enterovirus infections seems to have contributed to the decrease in the incidence of meningitis. Consequently, it can be concluded that the NPIs instituted to prevent the spread of the COVID-19 had some effect on reducing the incidence of meningitis, especially viral meningitis. Additional large-scale studies are needed, and the impact of the outbreak of COVID-19 on CNS infectious diseases other than meningitis needs to be studied.

## Data Availability Statement

The original contributions presented in the study are included in the article/supplementary material, further inquiries can be directed to the corresponding author/s.

## Ethics Statement

The studies involving human participants were reviewed and approved by the Institutional Review Board of the Catholic University of Korea. Written informed consent from the participants’ legal guardian/next of kin was not required to participate in this study in accordance with the national legislation and the institutional requirements.

## Author Contributions

JL, AC, KK, YK, and SK substantial contributions to the conception or design of the study and the acquisition, analysis, or interpretation of the data of the study. JL, AC, KK, JB, TE, IY, DY, SK, and YK critical revision for important intellectual content and agreement to be accountable for all the aspects of the study in ensuring that questions related to the accuracy or integrity of any part of the work are appropriately investigated and resolved and final approval of the version to be published. JL, KK, and YK critical revision for important intellectual content. JL and KK literature review. All the authors contributed to the article and approved the submitted version.

## Conflict of Interest

The authors declare that the research was conducted in the absence of any commercial or financial relationships that could be construed as a potential conflict of interest.

## Publisher’s Note

All claims expressed in this article are solely those of the authors and do not necessarily represent those of their affiliated organizations, or those of the publisher, the editors and the reviewers. Any product that may be evaluated in this article, or claim that may be made by its manufacturer, is not guaranteed or endorsed by the publisher.

## References

[1] GBD 2016 Neurology Collaborators. Global, regional, and national burden of neurological disorders, 1990–2016: a systematic analysis for the global burden of disease study 2016. *Lancet Neurol.* (2019) 18:459–80. 10.1016/S1474-4422(18)30499-X 30879893 PMC6459001

[2] GBD 2016 Meningitis Collaborators. Global, regional, and national burden of meningitis, 1990-2016: a systematic analysis for the global burden of disease study 2016. *Lancet Neurol.* (2018) 17:1061–82. 10.1016/S1474-4422(18)30387-9 30507391 PMC6234314

[3] Kim JW Chae SA Kim SY Lee NM Yi DY Yun SW Trends in pediatric meningitis in South Korea during 2009 to 2017: analysis of the health insurance review and assessment service database. *Ann Child Neurol.* (2021) 29:30–6. 10.26815/acn.2020.00178

[4] Funk S Gilad E Watkins C Jansen VA. The spread of awareness and its impact on epidemic outbreaks. *Proc Natl Acad Sci USA.* (2009) 106:6872–7. 10.1073/pnas.0810762106 19332788 PMC2672559

[5] Middeldorp M van Lier A van der Maas N Veldhuijzen I Freudenburg W van Sorge NM Short term impact of the COVID-19 pandemic on incidence of vaccine preventable diseases and participation in routine infant vaccinations in the Netherlands in the period march-september 2020. *Vaccine.* (2021) 39:1039–43. 10.1016/j.vaccine.2020.12.080 33478793 PMC7787078

[6] Hatoun J Correa ET Donahue SMA Vernacchio L. Social distancing for COVID-19 and diagnoses of other infectious diseases in children. *Pediatrics.* (2020) 146:e2020006460. 10.1542/peds.2020-006460 32879032

[7] Angoulvant F Ouldali N Yang DD Filser M Gajdos V Rybak A Coronavirus disease 2019 pandemic: impact caused by school closure and national lockdown on pediatric visits and admissions for viral and nonviral infections-a time series analysis. *Clin Infect Dis.* (2021) 72:319–22. 10.1093/cid/ciaa710 33501967 PMC7314162

[8] Kim MC Kweon OJ Lim YK Choi SH Chung JW Lee MK. Impact of social distancing on the spread of common respiratory viruses during the coronavirus disease outbreak. *PLoS One.* (2021) 16:e0252963. 10.1371/journal.pone.0252963 34125839 PMC8202938

[9] World Health Organization. *WHO-Recommended Standards for Surveillance of Selected Vaccine-Preventable Diseases.* Geneva: World Health Organization (2003).

[10] Hyndman RJ Khandakar Y. Automatic time series forecasting: the forecast package for R. *J Stat Softw.* (2008) 27:1–22. 10.18637/jss.v027.i03

[11] Kies KD Thomas AS Binnicker MJ Bashynski KL Patel R. Decrease in enteroviral meningitis: an unexpected benefit of coronavirus disease 2019 (COVID-19) mitigation? *Clin Infect Dis.* (2021) 73:e2807–9. 10.1093/cid/ciaa1881 33354704 PMC7799197

[12] Stoffel L Agyeman PKA Keitel K Barbani MT Duppenthaler A Kopp MV Striking decrease of enteroviral meningitis in children during the COVID-19 pandemic. *Open Forum Infect Dis.* (2021) 8:ofab115. 10.1093/ofid/ofab115 34183977 PMC8083471

[13] Luciani L Ninove L Zandotti C Nougairede A. COVID-19 pandemic and its consequences disrupt epidemiology of enterovirus meningitis, South-East France. *J Med Virol.* (2021) 93:1929–31. 10.1002/jmv.26785 33482046 PMC8014593

[14] Taha MK Deghmane AE. Impact of COVID-19 pandemic and the lockdown on invasive meningococcal disease. *BMC Res Notes.* (2020) 13:399. 10.1186/s13104-020-05241-9 32854773 PMC7450896

[15] Michos AG Syriopoulou VP Hadjichristodoulou C Daikos GL Lagona E Douridas P Aseptic meningitis in children: analysis of 506 cases. *PLoS One.* (2007) 2:e674. 10.1371/journal.pone.0000674 17668054 PMC1933255

[16] Logan SA MacMahon E. Viral meningitis. *BMJ.* (2008) 336:36–40. 10.1136/bmj.39409.673657.AE 18174598 PMC2174764

[17] Fellick JM Thomson AP. Long-term outcomes of childhood meningitis. *Hosp Med.* (2002) 63:274–7. 10.12968/hosp.2002.63.5.2019 12066345

[18] Erdem H Inan A Guven E Hargreaves S Larsen L Shehata G The burden and epidemiology of community-acquired central nervous system infections: a multinational study. *Eur J Clin Microbiol Infect Dis.* (2017) 36:1595–611. 10.1007/s10096-017-2973-0 28397100

[19] Korean Center for Disease Control and Prevention. *Coronavirus Disease-19 (COVID-19), Republic of Korea.* (2021). Available online at: http://ncov.mohw.go.kr/en/ (accessed December 30, 2021).

[20] Korean Center for Disease Control and Prevention homepage. *Coronavirus Disease-19 (COVID-19), Republic of Korea.* (2021). Available online at: http://ncov.mohw.go.kr/tcmBoardView.do?brdId=&brdGubun=&dataGubun=&ncvContSeq=355170&contSeq=355170&board_id=&gubun=ALL (accessed December 30, 2021).

